# Evaluation design of the patient-centred pathways of early palliative care, supportive ecosystems and appraisal standard (InAdvance): a randomised controlled trial

**DOI:** 10.1186/s12877-022-03508-3

**Published:** 2022-10-21

**Authors:** Junwen Yang-Huang, Ascensión Doñate-Martínez, Jorge Garcés, Maria Soledad Gimenez Campos, Raquel Valcarcel Romero, Maria-Eugenia Gas López, Adriano Fernandes, Mariana Camacho, Ana Gama, Sofia Reppou, Panagiotis D. Bamidis, Gordon Linklater, Frances Hines, Jude Eze, Hein Raat, on behalf of the InAdvance consortium, Michael Bennett, Vania Dimitrova, Nhu Tram, Marine Luc, Luis Fernández, Päivi Salminen, Vicent Blanes, Zoe Valero, Evdokimos Konstantinidis, Giuseppe Conti

**Affiliations:** 1 Department of Public Health, Erasmus Medical Center, P.O. Box 2040, 3000 Rotterdam, CA Netherlands; 2 Polibienestar Research Institute, University of Valencia, Valencia, Spain; 3 The University and Polytechnic La Fe Hospital of Valencia, 46026 Valencia, Spain; 4 The Medical Research Institute of the Hospital La Fe, 46026 Valencia, Spain; 5 Santa Casa da Misericórdia da Amadora (SCMA), Amadora, Portugal; 6 Lab of Medical Physics and Digital Innovation; and Special Unit for Biomedical Research and Education, School of Medicine, Aristotle University of Thessaloniki, Thessaloniki, Greece; 7 NHS Highland / Highland Hospice, Inverness, UK; 8 NHS Highand, Inverness, UK; 9 Department of Veterinary and Animal Science, Northern Faculty, Scotland’s Rural College, Inverness, UK

**Keywords:** Palliative care, Complex chronic conditions, Older persons, Early identification, Patient-centred care, Needs assessment, Cost effectiveness, Quality of life

## Abstract

**Background:**

Palliative care aims to contribute to pain relief, improvement with regard to symptoms and enhancement of health-related quality of life (HRQoL) of patients with chronic conditions. Most of the palliative care protocols, programmes and units are predominantly focused on patients with cancer and their specific needs. Patients with non-cancer chronic conditions may also have significantly impaired HRQoL and poor survival, but do not yet receive appropriate and holistic care. The traditional focus of palliative care has been at the end-of-life stages instead of the relatively early phases of serious chronic conditions. The ‘Patient-centred pathways of early palliative care, supportive ecosystems and appraisal standard’ (InAdvance) project implements and evaluates early palliative care in the daily clinical routine addressing patients with complex chronic conditions in the evolution towards advanced stages. The objective of the current study is to evaluate the acceptability, feasibility, effectiveness and cost-effectiveness of this novel model of palliative care in the relatively early phases in patients with chronic conditions.

**Methods:**

In this study, a single blind randomised controlled trial design will be employed. A total of 320 participants (80 in each study site and 4 sites in total) will be randomised on a 1:1 basis to the Palliative Care Needs Assessment (PCNA) arm or the Care-as-Usual arm. This study includes a formative evaluation approach as well as a cost-effectiveness analysis with a within-trial horizon. Study outcomes will be assessed at baseline, 6 weeks, 6 months, 12 months and 18 months after the implementation of the interventions. Study outcomes include HRQoL, intensity of symptoms, functional status, emotional distress, caregiving burden, perceived quality of care, adherence to treatment, feasibility, acceptability, and appropriateness of the intervention, intervention costs, other healthcare costs and informal care costs.

**Discussion:**

The InAdvance project will evaluate the effect of the implementation of the PCNA intervention on the target population in terms of effectiveness and cost-effectiveness in four European settings. The evidence of the project will provide step-wise guidance to contribute an increased evidence base for policy recommendations and clinical guidelines, in an effort to augment the supportive ecosystem for palliative care.

**Trial registration:**

ISRCTN, ISRCTN24825698. Registered 17/12/2020.

## Background

Population ageing is a long-term trend in Europe which began several decades ago [1]. In 2020, more than one fifth (20.6%) of the European Union (EU) population was aged 65 and over [2]. Moreover, the share of those aged 80 years or above is projected to more than double between 2016 and 2080 in the EU, from 5.4 to 12.7% [2].

With the predicted demographic shift, the prevalence of multi-morbidity, defined as the co-occurrence of two or more long-term conditions, will rise [3]. The prevalence of multi-morbidity among older persons (> 60 years) ranges from 55 to 98% worldwide [4]. For example, a study in Scotland reported that 65% of people aged 65–84 years and 82% of people aged 85 years and over are affected by chronic conditions and multi-morbidity [5]. The impact of chronic conditions and multi-morbidity may include impaired physical function, dependence, poor health-related quality of life (HRQoL), high care costs and decreased survival [6–8]. Palliative care is considered to be an appropriate way for pain relief, improvement of symptoms experience and well-being and enhancement of HRQoL of patients with chronic conditions. According to the World Health Organization (WHO), palliative care aims to improve the HRQoL of patients and their families who are facing the problems associated with life-threatening illnesses [9]. Palliative care prevents and relieves suffering by means of early identification, appropriate assessment and treatment of pain and other problems, whether physical, psychological, social or spiritual [9].

However, despite the growth in needs of palliative care services, it is estimated that only about 14% of people in need of palliative care are currently receiving it worldwide [10]. Most of the palliative care protocols, programmes and units are more focused on patients with cancer and their specific requirements and needs [11]. Patients with non-cancer chronic conditions may also have significantly impaired HRQoL and poor survival, but relatively often do not receive holistic and appropriate care according to their needs when compared to oncological patients [12, 13]. Furthermore, the core focus of palliative care providing relief from pain, physical symptoms, and psychosocial distress is at the end-of-life stages [14, 15]. As affirmed by the WHO, patients with chronic conditions may also benefit from palliative care actions in the early phases of their condition in conjunction with the specific life-prolonging treatments [16].

In addition, it is recognized that a relatively large share of healthcare resources is spent on the end-of-life care [17]. It is estimated that in the USA 25% of health-care expenditure is provided on patients’ last year of life [18]. Another study reported that in UK relatively 20% of hospital bed days are taken up by palliative care services [19]. However, the evidence base of cost-effectiveness evaluation to palliative care remains small [20–24]. One literature review reported that palliative care services mostly cost less relative to comparator groups (i.e. standard hospital setting, acute care services), and in most cases, the difference in cost is statistically significant [17]. More evidence on cost-effectiveness evaluation of palliative care is needed and can be served as drafting policy recommendations and clinical guidelines.

*The Patient-centred pathways of early palliative care, supportive ecosystems and appraisal standard* (InAdvance) project was set up to address the Horizon 2020 Call: SC1-BHC-23-2018 – ‘Novel patient-centred approaches for survivorship, palliation and/or end-of-life care’. The InAdvance project integrates early palliative care into daily clinical routine addressing patients with complex chronic conditions in evolution towards advanced stages.

## Aims and objectives

Within the InAdvance project at four locations a randomized trial will be performed lasting 18 months after the first intervention. In Valencia (Spain), Amadora (Portugal), Thessaloniki (Greece) and Highlands (UK) a novel model of palliative care will be implemented by integrating personalized pathways for palliative care that starts at an early stage of complex chronic conditions for older people (≥60 years old). The objective of our study is to evaluate acceptability, feasibility, effectiveness and cost-effectiveness of the novel model of palliative care in patients with chronic conditions.

In terms of effectiveness, self-reported outcome measures will be assessed with regard to both patients and informal caregivers/relatives to analyze the impact of the intervention on their wellbeing. The cost-effectiveness evaluation will assess whether there is ‘value for money’ and cost savings derived from the intervention. Finally, the implementation of the interventions process will be assessed at several times with the involvement of key healthcare professionals to identify facilitators and barriers of the implementation of the interventions.

We hypothesize that patients will have improved or maintained HRQoL and alleviated symptoms, and that multifaceted, complex needs including psychological, emotional and spiritual needs will be addressed. Informal caregivers/relatives will have improved or maintained HRQoL, increased social support, caring and coping skills and decreased caregiving burden. From the cost-effectiveness evaluation, we hypothesize that patients will have more efficient use of health care resources and contribute to a reduction of unnecessary costs.

## Methods/design

According to the needs identified and clinical pathways of each site, an intervention process was proposed based on the Consolidated Standards of Reporting Trials (CONSORT) flow diagram [25] aiming to: 1) identify patients who are at the advanced stage of their diseases, 2) assess potential palliative care needs of patients and their caregivers, and 3) define clinical pathways to address the identified needs. The NAT: PD (Needs Assessment Tool: Progressive Disease) [26] will be used to assess the needs of patients and their informal caregivers/relatives. The NAT: PD is a one-page assessment tool that consists of 21 items divided into four sections: 1) priority referral for further assessment; 2) patient wellbeing; 3) ability of caregiver or family to care for the patient; and 4) caregiver wellbeing. The level of concern is scored for each item as none, some or severe. The NAT: PD is developed to be used in both generalist and specialist settings that can facilitate decision-making on palliative care referrals to ensure that the identified needs can be better managed with appropriate services and support. The NAT: PD will be delivered either face-to-face or remotely via telephone or video, taking into consideration patient’s preferences and/or ability to visit the healthcare facility or respecting social distancing protocols established by national/regional health authorities under the current COVID-19 pandemic. The processing of personal data will be ensured in accordance with national and European regulations.

### Study design

The study has two stages. In stage one, a single blind randomised controlled trial will be conducted. Patients will be randomly assigned to two arms – the ‘Palliative Care Needs Assessment (PCNA)’ arm and the ‘Care-as-Usual’ arm. Primary intervention using the NAT:PD with repeated measures of the variables will be carried out in the PCNA arm. In stage two, patients and their informal caregivers/relatives assigened to the PCNA arm will benefit from a range of supportive programmes. These feasibility pilots in stage two will be adapted to the particularity of each clinical site. In Stage Two, in the ‘Care-as-Usual’ arm, the ‘usual care’ will be continued. The trial is registered at Cochrane Library: ISRCTN24825698.

**Fig. 1:**
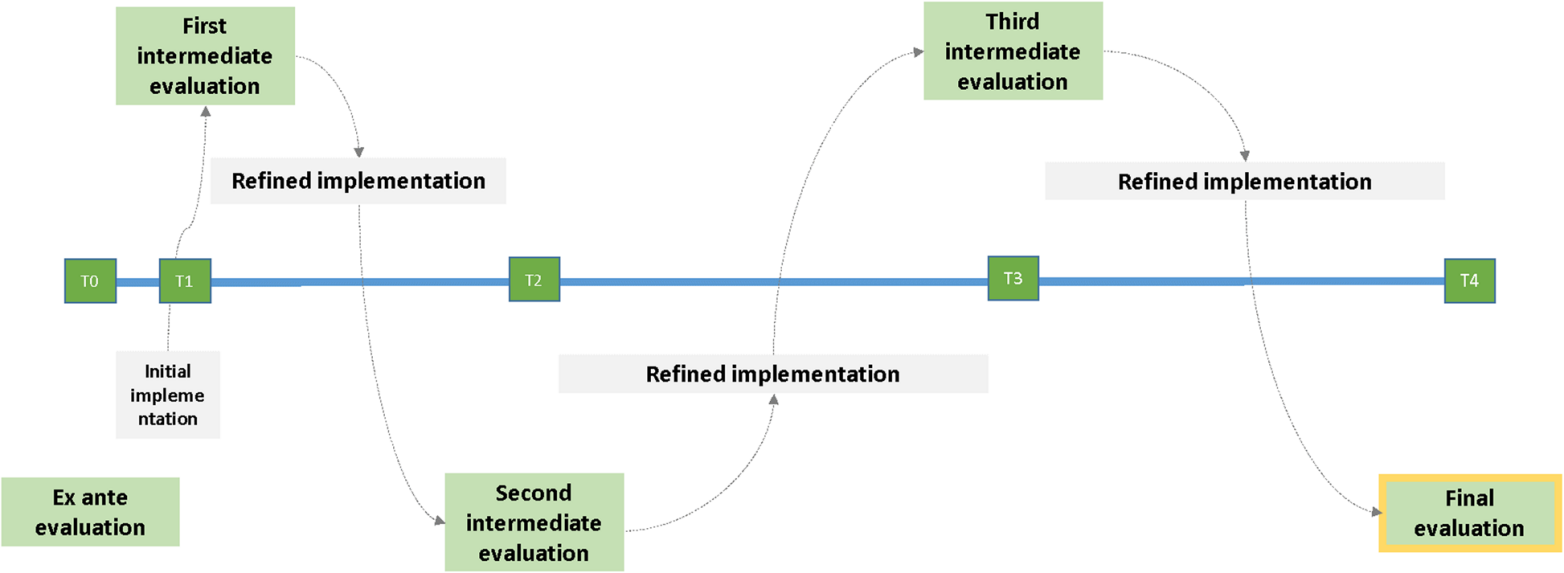
Trial outcome evaluation schedule

The evaluation study of the InAdvance project includes a formative evaluation approach, which provides information regarding feasibility at real-time implementation (Fig. 1). After patients’ recruitment in the study, baseline data (T0) will be collected. Three intermediate evaluations will be performed at week 6 (T1), month 6 (T2) and month 12 (T3) after the implementation of the intervention. A final evaluation will be performed on month 18 (T4).

### Study setting

The InAdvance intervention is a population-base prospective cohort study conducted in four settings. At the locations in Spain patients with multi-morbidity will be included. At the location in Greece patients with respiratory disease, patients with heart failure and patients with frailty will be included. At the location in Portugal and the UK patients with multi-morbidity or severe chronic obstructive pulmonary disease (COPD) will be included.

### Eligibility criteria and recruitment

Men and women of at least 60 years of age diagnosed with complex chronic conditions with a potential progressive course are eligible for the study, as well as their caregivers. A detailed description of the inclusion and exclusion criteria is shown in Table 1.

**Table 1:** Inclusion and exclusion criteria related to patients for the needs assessment trail

Inclusion criteria	Exclusion criteria
• Participants with capacity to provide informed consent	• Individuals unable to provide written informed consent
• **Spanish, Portuguese and Greek sites:** No more than mild cognitive decline (≥18 points) on the Mini Mental State Examination (MMSE) [27] or up to 4 errors on the Pfeiffer Test [28] **UK site:** If the participant’s clinical record includes documented evidence of cognitive decline, participants should demonstrate no more than mild cognitive decline at screening (must score ≥ 18 points on MMSE)	• **Spanish, Portuguese and Greek sites:** Older adults diagnosed with cancer **UK site:** Older adults diagnosed with active cancer (excluding basal cell carcinoma and squamous cell carcinoma of the skin)
• Males and females	• Individuals currently enrolled on any other research which might interfere with the results and the procedure of the study
• Participants aged 60 years or older at the time of randomisation	• Individuals under the age of 60 years
• **UK, Portuguese and Greek sites:** Participants that have a clinical diagnosis of severe COPD or severe respiratory illness OR **UK site:** Participants with at least two co-occurrent chronic conditions (multi-morbidity), at least one of them being in a severe stage as determined by the patient’s clinical care team OR **Spanish and Portuguese sites:** Participants with at least two co-occurrent chronic conditions (multi-morbidity), at least one of them in a severe stage according to the specific criteria of severity and progression of disease of the NECPAL tool [29] OR **Greek site:** Participants that have a clinical diagnosis or severe respiratory illness and multi-morbidities (participants with severe heart failure, diabetes, lipidemia, hypothyroidism, or arthritis)	• Individuals who, in the opinion of the research team, may face physical or psychological risk/harm due to the invite participation or the enrolment on the study (i.e. subjects that are not aware of the severe prognosis, unstable clinical conditions)
• **Portuguese and Greek sites:** Owning a Smartphone and the ability to use it (or an informal care giver having the ability to use it)	
• **Spanish, Portuguese and Greek sites:** No previous referral to specialist Palliative Care **UK site:** No referral to specialist Palliative Care in the 2 years prior to randomisation	
• Be aware of their diagnosis and prognosis	
• Willingness to participate in the study	
• Not currently enrolled in other research studies	

### Sample size considerations

Assuming a statistical power of 95% and an effect size of 0.20 regarding continuous outcome measures between intervention and control group with an alpha risk of 0.05, the sample size is estimated to be 320 participants; that is 80 at each of the four sites with 40 participants in the intervention group and 40 in the control group. In the study, for each patient participating in the study, it is expected to include one informal caregiver (e.g. a family member) who provides informed consent to join the study. Furthermore, for the formative evaluation, 10–15 health care professionals will be recruited to participate in the study at each study site.

### Outcome measures and data collection

Data will be collected using self-reported questionnaires translated into the official language of each country. Validated translations will be used. When needed, the questionnaire will be translated with the forward-backward translation technique [30] and tested in each setting after being reviewed and adapted. Before the start of the study, questionnaires will be pilot-tested in all participating. The instruments used to measure the outcomes are described below.

Patient-reported outcomes to evaluate trial effectiveness includes HRQoL, intensity of symptoms, functional status, emotional distress, perceived quality of care and adherence to treatment. HRQoL is measured with the EQ-5D-5L instrument [31], which includes: mobility, self-care, usual activities, pain/discomfort and anxiety/depression. Intensity of symptoms is measured with the Palliative Care Outcome Scale (POS) [32]. The POS measures patients’ physical, psychological, emotional and spiritual symptoms, and is suitable for use in both specialist palliative care and non-specialist palliative care settings. Functional status is measured via the Palliative Performance Scale Version 2 (PPSv2) that includes five domains: ambulation, activity & evidence of disease, self-care, intake and conscious level [33]. Emotional distress is assessed with the Hospital Anxiety and Depression Scale (HADS) among patients [34]. It is a two-dimension scale developed to identify depression and anxiety among physically ill patients and the general population valid at hospital and in community settings. Perceived quality of care is assessed through a 5-point Likert scale related to communication, information provided, personalized care, family-centred and overall satisfaction. Adherence to treatment is assessed through the Medical Outcomes Study (MOS) [35].

Emotional distress and perceived quality of care will also be assessed among relatives or informal caregivers. In addition, caregiving burden will be measured among relatives or informal caregivers through the brief version of Zarit Burden Interview (ZBI) [36]. ZBI aims to evaluate the perceived impact of providing care on aspects such as the caregiver’s health, personal and social life, financial situation, emotional wellbeing and interpersonal relationships [37, 38].

For the purpose of the cost-effectiveness analyses, three categories of costs will be measured: the intervention costs, other healthcare costs and informal care costs. The intervention costs concern the resource units consumed (e.g. minutes spent by health and social care professionals, diagnostic procedures and consumables) and their unit costs, which will be collected by interviewing professionals as well as from Patient- or Administrative Data Management Systems. Special attention will be paid to overhead costs as they represent between 35 and 40% of intervention costs [39]. Other healthcare costs will be measured with the Medical Consumption Questionnaire (MCQ) [40], which will be completed by patients. The MCQ includes questions related to frequency of visits to healthcare professionals, hospital’s accident and emergency department and frequency and duration of hospital admissions. Informal care costs will be determined by the number of hours taking care of the patient with corresponding hourly productivity costs (Valuation of Informal Care Questionnaire (VICQ) [40]).

In terms of implementation, healthcare professionals involved in the implementation of the interventions will be invited to participate in personal interviews, focus groups and to complete questionnaires with a 5-point Likert scale. Items of those interviews and questionnaires are based on the Consolidated Framework for Implementation Research (CFIR). This framework guides diagnostic assessments of implementation context, evaluates implementation progress and helps explain findings in studies or quality improvement initiatives [41]. Both qualitative and quantitative information regarding receptivity, potential barriers and facilitators to implement the intervention will be gathered.

The baseline questionnaire also includes items regarding the socio-demographic characteristics of the patients, relatives/informal caregivers and healthcare professionals (see Table 2). Patients’ medical histories are also included in the baseline questionnaire. Table 3 presents the different measures to be used among the different end-users involved in the randomized controlled trials.

**Table 2:** Socio-demographic and general characteristics

Variables	End-user
Age	Patient, informal caregiver, healthcare professionals
Sex	Patient, informal caregiver, healthcare professionals
Marital status	Patient
Level of education	Patient, informal caregiver, healthcare professionals
Ethnic background	Patient, informal caregiver
Professional category	Patient, informal caregiver
Relationship with the patient	Informal caregiver
Caregiving skills	Informal caregiver
Years of experience in caregiving	Informal caregiver
Preferences for place of care	Patient, informal caregiver, healthcare professionals
Preferences for place of death	Patient, informal caregiver, healthcare professionals
Active diagnosis (main diseases)	Patient
Time since initial diagnosis (main diseases)	Patient
Number of prescribed drugs	Patient
Type of prescribed drugs	Patient
Position	Healthcare professionals
Years of experience in general healthcare practice	Healthcare professionals
Years of experience in palliative care	Healthcare professionals
Previous training in palliative care	Healthcare professionals

**Table 3:** Indicators of the feasibility, effectiveness and costs of the intervention

End user	Outcomes	Instrument	Moment
Patient	Health-related quality of life	EQ-5D-5L	T0, T1, T2, T3, T4
	Intensity of symptoms	POS	T0, T1, T2, T3, T4
	Functional status	PPSv2	T0, T1, T2, T3, T4
	Emotional distress	HADS	T0, T1, T2, T3, T4
	Quality of care	Short set of items	T0, T1, T2, T3, T4
	Other healthcare costs	MCQ	T0, T1, T2, T3, T4
	Adherence to treatment	MOS	T1, T2, T3, T4
Informal caregivers/ Relatives	Health-related quality of life	EQ-5D-5L	T0, T1, T2, T3, T4
	Emotional distress	HADS	T0, T1, T2, T3, T4
	Caregiving burden	Brief ZBI	T0, T1, T2, T3, T4
	Informal care costs	VICQ	T0, T1, T2, T3, T4
	Quality of care	Short items	T1, T2, T3, T4
Healthcare professionals	Feasibility, acceptability, adoption, appropriateness of the intervention	CFIR-based interview and questionnaire	T0, T1, T2, T3, T4
	Fidelity	Short set of questions based on the interventions’ components	T1, T2, T3, T4
	Intervention costs	Uniform reporting template; interview	T1, T2, T3, T4

### Data management and statistical analysis

The University of Valencia (UVEG) and the Erasmus Medical Center (Erasmus MC) are responsible for the analysis and reporting. UVEG, Erasmus MC and together with all clinical sites are responsible for the data manegement. All data will be gathered through the CASTOR eCRF system, which will be made available to the consortium.

Descriptive statistics will be carried out to summarize characteristics of participants in each clinical site and in the total study population. Tests for normal distribution of the continuous outcome measures will be performed using the Kolmogorov–Smirnov test. The outcome measures will be compared within each group (intervention group and control group) at different measurement times (i.e. T0-T1; T1-T2; T2-T3; T3-T4; T1-T4; T0-T4, etc.). A multilevel modelling approach will be used to examine differences in the outcome measures between the intervention and control group, taking into account the clustering effects at the clinical site level. The relationship between the outcome measures and independent variables will be assessed using Pearson correlation (for continuous variables) and Pearson Chi-square test (for categorical variables). Comparison between the intervention group and the control group at each evaluation moment will be conducted by including an interaction between the treatment groups and time in the multilevel model.

The cost-effectiveness analysis will be conducted from a societal perspective and on a within-trial horizon. The incremental cost-effectiveness ratio (ICER) will be calculated and the degree of uncertainty for costs and effectiveness and the cost-effectiveness ratio will be examined on the ICER by using non parametric bootstrapping. In addition, an acceptability curve will be generated to indicate the probability that the intervention has lower incremental costs per quality adjusted life year (QALY) gained than various thresholds for the maximum willingness to pay for an extra QALY.

In all cases, *p*-values < 0.05 will be considered statistically significant. All analyses will be carried out using the statistical package SPSS v26 or similar statistic software packages.

## Discussion

This study aims to evaluate the acceptability, feasibility, effectiveness and cost-effectiveness of early palliative care interventions in older European persons with complex chronic conditions. This is done using a randomised controlled trial design in four European cities: Valencia (Spain), Amadora (Portugal), Thessaloniki (Greece) and Highlands (UK). In accordance with the local clinical pathways and resources, individualized care pathways will be designed for each city. Carrying out individualized care pathways will foster that the InAdvance interventions are feasible and sustainable beyond the project life. This could also help facilitate future implementation into different key care levels: hospice care, primary care and community and social care. The development of early palliative care intervention was based on the experiences and preferences of a diverse group of stakeholders (older persons, informal caregivers/relatives and healthcare professionals), and was oriented by the end-users which could generate an adequate compliance to the intervention [42].

This study has several strengths. As far as we know, this is one of the first European studies that aims to integrate early palliative care in the daily clinical routine specifically addressing on patients with non-oncological complex chronic conditions in different European settings. Studying the feasibility of early palliative interventions is relevant, specially due to the stigma associated with palliative care or with end- of- life care. An early integration of palliative care among oncological patients has been proved feasible and well-accepted both for patients and their relatives [43]. Additionally, according to a recent policy brief from the European Observatory on Health Systems and Policies [44], a skilled assessment of patients’ needs and the support from them and their relatives can improve the experiences for both and may reduce costs of care. In this line, InAdvance intervention aims to timely detect palliative needs and to assist them in accordance with patients’ and their relatives’ goals and desires. In addition, a set of technology-based secondary interventions will also be deployed as a manner of feasibility/proof-of-concept studies. These technologies would also be a relevant asset in the support, monitoring and palliation of physical, emotional and social needs of older patients [45] as well as an effective learning method for healthcare professionals [46].

The proposed study has some limitations and we expect to encounter some challenges. There is an absence of a uniform definition on early palliative care in Europe. No complete agreement has been reached about which model of care and interventions should be early integrated in the course of chronic conditions with the aim to improve the quality of life of patients and their families [47, 48]. The approaches and interventions implemented differ widely depending on the diseases considered, the care settings, the outcome measures and the geographic, social and cultural contexts of both patients and medical systems [49, 50]. The InAdvance interventions will be developed based on specific core elements of palliative care, taking into consideration various stakeholders’ perceptions, services and resources available for the palliative care and local culture and legal framework. The InAdvance aims to provide insights into the key components that could foster the timely integration of palliative care in the trajectory of severe chronic conditions. Participation of older persons with complex chronic conditions may be a challenge, especially during the COVID-19 pandemic. To increase participation, remote recruitment, enrollment, visits and data collection procedures, such as phone or videoconference calls will be considered increasing the efficiency, reducing costs and mitigating the risk of infection especially among older populations with chronic conditions [51].

As the growth of the older population will pose a challenge for the healthcare system, providing more effective care is necessary. A patient-centred and early implementation of palliative care may adequately address the needs of patients with chronic conditions. The InAdvance project will further elucidate whether such an approach could be effective and feasible for older population in different settings and identify potential effective elements of early palliative care, with the aim to define mechanisms for establishing a supportive ecosystem for palliative care.

## Data Availability

The datasets generated and/or analysed during the current study are not publicly available. The availability of data and materials will be discussed between partners and will be available on reasonable request.
